# Conductive organic electrodes for flexible electronic devices

**DOI:** 10.1038/s41598-023-30207-9

**Published:** 2023-03-13

**Authors:** Amrita Chakraborty, Daniel Herrera, Payton Fallen, Daniel Hall, Nicholas Bampton, Thomas Olivero, Marius Orlowski

**Affiliations:** Bradley Department of Electrical and Computer Engineering, Virginia Tech, Blacksburg, VA 24061 USA

**Keywords:** Biotechnology, Engineering, Materials science, Nanoscience and technology

## Abstract

The paper reports on a novel process flow to manufacture conductive organic electrodes from highly conductive doped PEDOT:PSS polymer films that can be patterned and display a good adhesion to oxidized Si wafers as well as to flexible substrates, such as Mylar. Among other results, it is shown that multiple depositions of PEDOT:PSS increase the electrical conductivity by more than two orders of magnitude without increasing the film thickness of PEDOT:PSS significantly. An exponential dependence between sheet resistance and the number of PEDOT:PSS coatings has been found. The electrical conductivity of PEDOT:PSS can be increased by another two orders of magnitude doping with Cu nanoparticles when coated on the surface of a soft-baked PEDOT:PSS film. It is found, however, that both kinds of conductivity enhancement are not additive. Adhesion of PEDOT:PSS to oxidized Si wafers and BoPET (Mylar) has been ensured by applying an oxygen plasma cleaning step before spin coating. The manufactured high-conductivity PEDOT:PSS film can be patterned using a sacrificial metal layer with subsequent etching of PEDOT:PSS in oxygen plasma, followed by the removal of the patterned segments of the sacrificial metal layer in an aqueous acid solution.

## Introduction

Among the promising candidates for organic highly conductive films such as polyacetylene, polypyrrole (PPy), polyaniline (PANI), and poly(3-hexylthiophene) polymer (P3HT), poly 3,4-ethylene dioxythiophene: polystyrene sulphonate (PEDOT:PSS)^1^ has attracted a lot of attention in various fields of application. The advantages of PEDOT:PSS include, mechanical flexibility and integrity, good thermal stability, high transparency in the visible range, light weight, easy processing and deposition by spin coating, spray coating, and ink-jet printing, and a variety of methods to increase conductivity by several orders of magnitude (still under active research), and a work function between 4.5 eV and 5.2 eV roughly comparable with work function of Cu of 4.7 eV, which is favorable for applications in hybrid crystalline silicon solar cells^2^.

Successful application of PEDOT:PSS has been reported in optoelectronic devices as a transparent and flexible electrode^3–8^, in next-generation photovoltaics^9^,^10^ in energy devices, and in functional packaging layers^11,12^. PEDOT:PSS conductive layers have successfully supplanted indium-tine oxide (ITO) films^13–15^ as a transparent conductor. The high transparency in the visible range of PEDOT:PSS facilitates the application of PEDOT:PSS in semitransparent photovoltaics. The group of F. Zhang^16^ reported fabrication of semitransparent polymer solar cells (PCS) with 9.40% power conversion efficiency and 24.6% visible transmittance and optimized PCS with a power conversion of 15.6% with high transmittance in the visible light range and low transmittance in the near infrared range^17^. Z. Xie et al.^18^ have demonstrated highly conductive PEDOT:PSS transparent electrode prepared by a post-spin-rinsing method for polymer solar cells. In charge extraction and injection devices, PEDOT:PSS has been used as an effective buffer layer^19–21^. PEDOT composites serve as a redox-active component in energy storage cells^22,23^. Additionally, bioelectronic devices^24,25^, and thermoelectric materials^26^ have taken advantage of PEDOT:PSS. Highly conductive PEDOT:PSS films have been reported for ITO-free liquid crystal display^27^.

Despite all of its advantages, the electrical conductivity of pristine PEDOT:PSS films is on the order of 0.1 S cm^−1^, which may be much larger than that of metal oxides such as TaO_x_ of 10^−6^ S cm^−1^ but is still significantly lower than the conductivity of metals such as Ta of roughly of 7 × 10^4^ S cm^−1^. Many different methods have been identified to enhance the electrical conductivity of PEDOT:PSS^27^. Before giving a brief review of the enhancement techniques, a deeper look into the properties and morphology of the PEDOT:PSS films is warranted to inform possible doping methods^1^. PEDOT:PSS consists of two components: the conductive PEDOT and the insulating PSS, whose oligomers entangle the PEDOT oligomers. The hydrophobic and water-insoluble PEDOT grains stay in the core surrounded by a shell of the hydrogen sulfate groups of the hydrophilic PSS chains which attach to the core surface and form a micelle structure. The thickness of the grain boundary is 30 Å–40 Å^28^. The positively charged PEDOT core is electrostatically bound to a PSS polyanion. During the deposition, due to its hydrophobic nature, PEDOT tends to stay away from water and settles at the bottom of the film while the hydrophilic and hygroscopic PSS strings, which are attracted to water, occupy the top portions of the film^29^. Thus, in wet films a phase separation occurs between the PEDOT-rich bottom and PSS-rich top The subsequent thermal annealing at temperatures between 90 °C and 130 °C leads to improvement of electrical conductivity due to the decreased water absorption and increased density of PEDOT grains, being insufficient for remixing the two phases. The phase separation leads to two different lateral and perpendicular conductivities of the PEDOT:PSS films. While the lateral electric conductivity occurs via hopping of charge carriers, the perpendicular conductivity (roughly 3 orders of magnitude smaller) is dominated by space charge effects. After the spin coating deposition PEDOT:PSS consists of horizontal layers of flattened PEDOT grains separated from one another by quasi continuous barriers of PSS ribbons.

Based on those properties of PEDOT:PSS films, there are three major strategies for enhancing its electrical conductivity: (a) increase the concentration of PEDOT grains while at the same time decrease the concentration of PSS, (b) to screen the electrostatic attraction between PEDOT and PSS and thus facilitate the electron hoping between π bonds across the carbon skeleton, and (c) by introducing nanoparticles or nanocomposites to bridge the distance between PEDOT islands within the PSS bulk. An excellent review of those approaches has been given by the Sarifuddina group^1^. X. Zhang et al.^30^ have manufactured inkjet-printed thin films of PEDOT:PSS doped with silver nanoparticles with excellent electrical and optical properties. Patil et al.^31^ also reported an enhanced conductivity of PEDOT:PSS by doping it with silver nanoparticles. R.-C. Zhang et al.^32^ have demonstrated a one-step synthesis process for gold nanoparticles—PEDOT:PSS nanocomposites and their successful application as an alkaline direct ethanol fuel cell catalyst. O. Ghazy et al.^33^ have incorporated silver particles prepared by gamma radiation to enhance the conductivity of PEDOT:PSS for the application of organic solar cells. L. Pham et al.^34^ have demonstrated improved polymer conductivity when doped with Cu nanoparticles (NP).

The final step in the process integration to manufacture organic electrodes is the patterning of conductive polymers. The patterning of organic polymer films has been performed by various methods—vapor deposition through shadow masks, by ink-jet printing, soft and hard imprint lithography, and conventional photolithography^35,36^. Ink-jet printing exhibits excellent roll-to-roll process capabilities and is the technique of choice for patterning of polymeric materials. However, its resolution is limited to 10–20 μm^36,37^. Shadow-mask deposition is an ubiquitous technique for small-molecule patterning, but also suffers from resolution limitations in the range of around 25–30 μm^38^. Moreover, shadow-mask deposition requires a high-vacuum chamber. The smallest feature resolution down 10 nm has been demonstrated by imprint lithography^39,40^ However, this technique is quite expensive and available only in highly specialized laboratories. Furthermore, in all of the aforementioned methods, challenging registration of related patterns on separate photomasks is a thorny issue and renders fabrication of multilayer devices difficult. Photolithography remains the most attractive patterning technique for the patterning of inorganic electronic materials as it is a standard patterning method in modern silicon-based semiconductor industry. However until recent, photolithography has made little inroads in patterning of PEDOT:PSS, due to a lack of chemical compatibility with PEDOT:PSS. Traditional photolithography leads to a chemical deterioration of active organic materials upon exposure to process solvents for lithography^39^. Recently, Ouynag et al.^40^ have reviewed current PEDOT:PSS patterning approaches using a modified photolithography processes. Through properly selected materials, insertion of sacrificial protective layers and etch processes, PEDOT:PSS can be patterned while avoiding this deterioration. Ouyang et al.^40^ have demonstrated a process in which photoresist is deposited and patterned on top of a sacrificial silver layer, which is covering PEDOT:PSS layer. Next, using suitable silver etchant such as nitric acid, the silver interlayer is selectively removed, exposing PEDOT:PSS segments that can be etched by oxygen plasma. After stripping the photoresist and etching the remnant silver islands, PEDOT:PSS patterns can be manufactured on Si wafer substrates. Taylor et al.^41^ have also shown that even nanoscale PEDOT:PSS patterns can be realized by utilizing orthogonal solvents along with corresponding photoresist, and by introducing a new set of benign processes that involve new specially tailored photopolymers. Finally, it has been demonstrated that a picosecond laser direct ablation with 355 nm and 1064 nm wavelength pulses can be successfully used^42–44^ to pattern PEDOT:PSS.

In summary, the important challenges with using PEDOT:PSS as for highly conductive films, remain to be patterning, adhesion to the substrate, material compatibility, and reaching conductivity in excess of 10^3^ S cm^−1^.

## Device fabrication

A commercial dispersion of PEDOT:PSS (1.3wt% dispersed in H_2_O, conductive grade) in water was purchased from Sigma-Aldrich and used to deposit organic films. The dispersed solution has a composition of 0.5wt.% PEDOT and 0.8wt.% PSS. This PEDOT:PSS dispersion was spin-coated onto cleaned substrates. The cleaning was performed by first placing cleaved rectangular pieces of oxidized Si wafers and biaxially-oriented polyethylene terephthalate (BoPET called also Mylar) of size larger or equal to 1.5 cm × 1.5 cm into an acetone bath and sonicated for at least 10 min. Subsequently, the substrate pieces were then transferred into an isopropyl alcohol (IPA) bath and sonicated for further 10 min. The substrates were then subsequently rinsed with deionized (DI) water followed by a blow dry with nitrogen and then placed onto a prewarmed hot plate (110 °C) for 1–2 min to dehydrate.

Initial spin-coating of PEDOT:PSS did not succeed at all—no PEDOT:PSS could be detected on the wafer. The solution to the non-uniform coverage of PEDOT:PSS onto the substrates was the introduction of an O_2_ plasma cleaning step immediately before spin-coating. Oxygen plasma in the SAMCO RIE chamber has been used with the power of 50W for 30 s at an oxygen flow rate of 20 sccm. It was ensured that the reflective power is less than 1 Watt. (In the case of mylar, the substrate pieces were taped to an oxidized Si wafer carrier in order to prevent flipping over while the RIE chamber is outgassing.) Immediately after the plasma treatment, the samples were moved to the spinner. The best uniformity of the PEDOT:PSS films was achieved with a two-step recipe:

- Step 1: Spin speed 500 rpm, ramp 200 rpm/s, duration 10 s.
- Step 2: Spin speed 2000 (1500)rpm, ramp 500 rpm/s, duration 45 s.

The above two cycles were modified from a standard photoresist deposition. The ramping parameters and spinning times have been varied to obtain optimum uniformity results. The final thickness of the PEDOT:PSS films for the maximum spin speeds of 1500, 2000 and 3000 rpm were 65, 56 and 29 nm, respectively.

After spin-coating, the samples were soft-baked on a hot plate at temperature 120 °C for 10 min to solidify the films. A lower temperature of 95 °C has been used for the soft bake, compatible with the photoresist soft bake, but no difference in conductivity was observed.

The thickness of PEDOT:PSS films was measured with a Dektak profilometer and atomic force microscope (AFM), whose measurements were within ± 1 nm of each other. The uniformity of the PEDOT:PSS film both on oxidized Si wafers and on Mylar was also determined to be + /− 2 nm. In the case of Mylar, it was found that for reliable thickness measurements, the area of the Mylar pieces must be at least 1.5 cm × 1.5 cm. Otherwise, the PEDOT:PSS exhibited rough edges and pooling of the material in the corners of the substrate. After patterning the PEDOT:PSS, the thickness of the PEDOT:PSS lines were ~ 4 nm, and lower in the center than at the edges, caused by the etching of Ag sacrificial layers in HNO_3_ solution.

The main body of research was devoted to the enhancement of electrical conductivity of the PEDOT:PSS films. The pristine PEDOT:PSS at 2000 rpm exhibited a sheet resistance ${R}_{S}$ of about 1MΩ/sq at a film thickness of 56 nm. According to the Eq. (1)

1 $$\sigma =\frac{1}{{R}_{S}\times t}.$$

The electrical conductivity σ is the inverse of the product of sheet resistance and film thickness. Thus, a sheet resistance of 1MΩ /sq at a film thickness of t = 56 nm corresponds to σ = 0.18 S/cm.

A number of different methods of enhancing PEDOT:PSS conductivity have been undertaken, including multiple spin coatings and doping the film with either Cu nanoparticles. Two kinds of Cu NP with 25 nm and 60 − 80 nm particle size from Sigma Aldrich^45^ were used. Since Cu NP are easily oxidized in aqueous solutions at ambient temperatures, suspending Cu NP directly in the aqueous PEDOT:PSS dispersion was avoided.

Instead, both sizes of the Cu nano-powder (NP) were separately dispersed in ethanol at two different concentrations: 0.2, and 0.5 mg/ml. A solution of 10 ml ethanol with the mentioned concentrations of Cu NPs for each kind was prepared. This solution was then stirred for an hour followed by ultrasonication for another 1 h. The Cu-ethanol dispersion was then spin coated on top of a PEDOT:PSS layer using the same spin coating recipe as for the PEDOT:PSS.

In order to further prevent oxidation of the Cu NP, the as-spun film was not soft baked. Instead, the sample was allowed to air dry for at least 5 min, during which the excess ethanol evaporated. The spinning of the Cu dispersion onto PEDOT:PSS did not measurably increase the thickness of the PEDOT:PSS film.

In order to pattern PEDOT:PSS using conventional lithography, the PEDOT:PSS film must be protected from exposure to the UV light during photoresist exposure. Additionally, PEDOT:PSS is damaged during photoresist deposition, development, and the removal step due to interaction with chemical reagents, especially the aqueous alkaline solutions used widely in conventional photolithography. Due to the acidity of PEDOT:PSS films, the crosslinking of traditional acid-sensitive photoresists is also adversely affected. Furthermore, acidic PEDOT:PSS films may cause decomposition of a positive photoresist layer. Chromium (Cr) or silver (Ag) thin films deposited by Physical Vapor Deposition (PVD) with a thickness of 100 nm were used as a protective layer on top of the photoresist during patterning. The Ag layers were superior to Cr layers, as the latter displayed partial peeling when rinsed in DI water, indicative of poor adhesion to the PEDOT:PSS film. After a photolithography step and development of the positive resist AZ 5214E-IR using the developer AZ300MIF, the exposed Ag regions were etched in a 5:8 solution of HNO_3_:H_2_O for about 11–12 s, until visible removal of Ag was observed. After the 12 s HNO_3_ etch the sample was rinsed twice in DI water. The uncovered PEDOT:PSS layer was removed in a Samco RIE chamber using oxygen plasma at 50W for 30 s at an O_2_ flow rate of 20 sccm. In the case of mylar, the rectangular pieces of mylar were taped down to the chuck to prevent lifting while the chamber is outgassing. Subsequently, the sample was sprayed with acetone for 30 s to remove the remaining photoresist material. Lastly, the remaining sacrificial Ag layer is removed when the samples are dipped in the HNO_3_:H_2_O (5:8) solution for 11–12 s, thus uncovering the patterned PEDOT:PSS.

**Figure 1:**
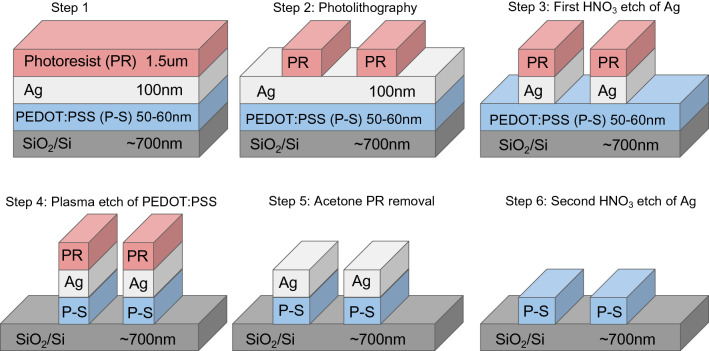
Photolithography process flow to pattern PEDOT:PSS films on oxidized Si wafers and on Mylar flexible substrates using Ag as sacrificial layer to protect PEDOT:PSS from damage during the photolithography exposure and photoresist ashing.

**Figure 2:**
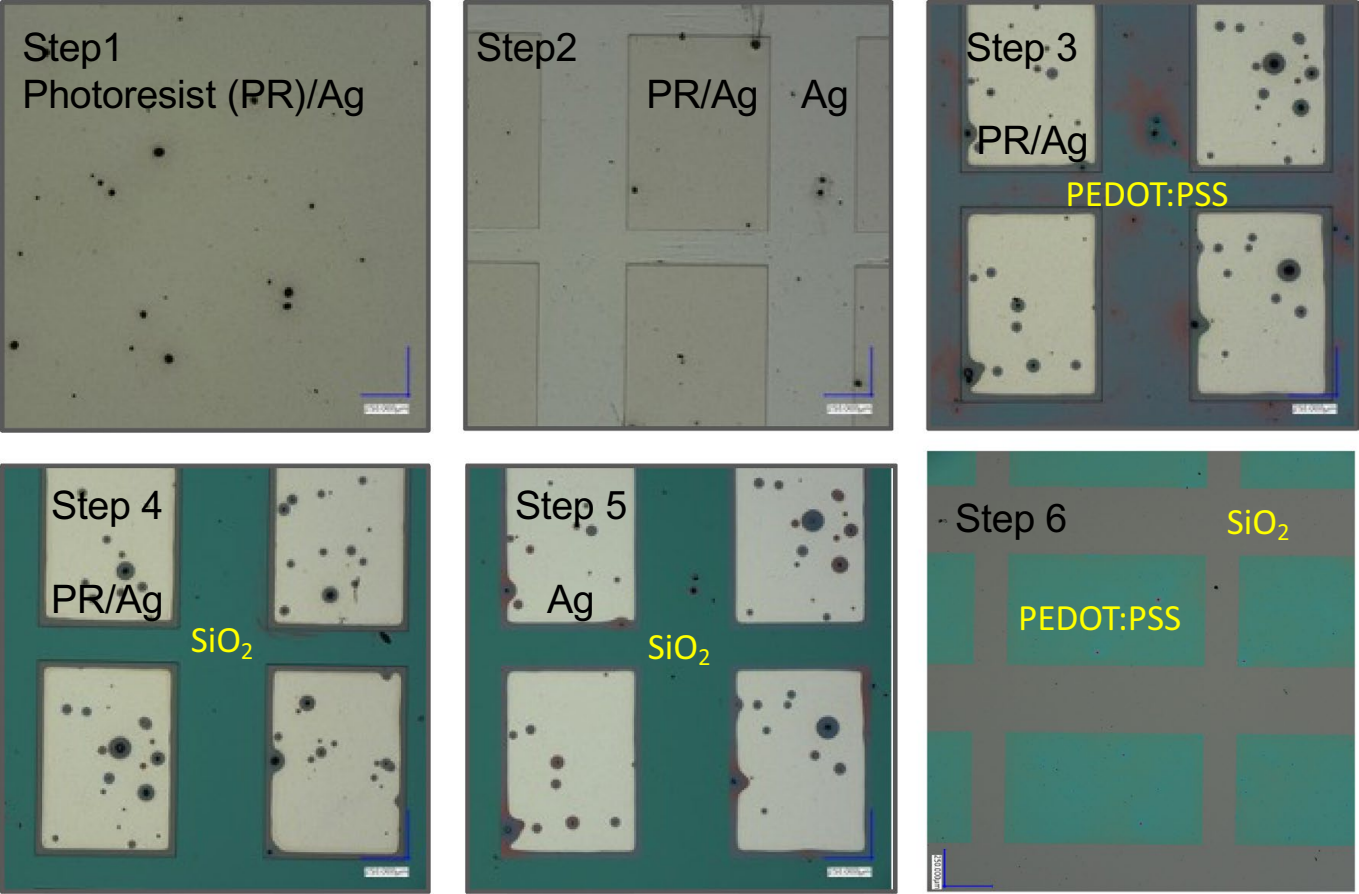
Optical pictures of the patterning process of PEDOT:PSS on an oxidized Si wafer. The six steps correspond to the six steps of the photolithography process flow shown in Fig. 1.

**Figure 3:**
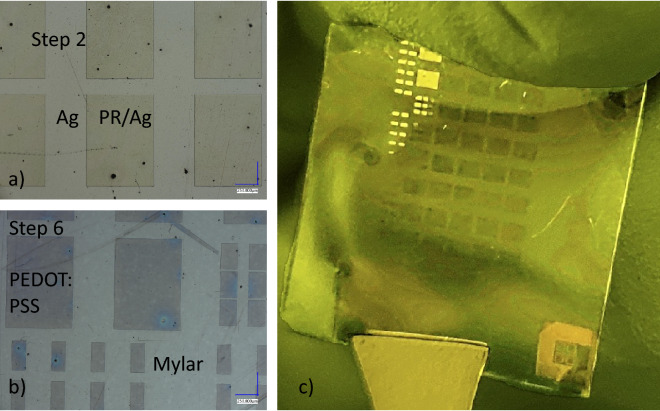
PEDOT:PSS patterning on Mylar flexible substrate. (**a**) patterning of the photoresist of the layer stack PR/Ag/PEDOT:PSS/Mylar. (**b**) The final PEDOT:PSS patterns on Mylar. (**c**) 2 × 2 cm piece of Mylar with PEDOT:PSS patterns on it.

The process flow of the photolithographic patterning of PEDOT:PSS is shown in Fig. 1. In Fig. 2 optical pictures of the patterns on oxidized Si wafers after every process step explained in Fig. 1 are shown. Figure 3 shows optical picture of the same photolithography patterning process on Mylar flexible substrate. Here, the optical pictures of step 2 and 6 are shown together with a piece of flexed Mylar with PEDOT:PSS patterns.

## Results and discussion

The most surprising result of conductivity enhancement of the PEDOT:PSS has been achieved by multiple spin coatings of the PEDOT:PSS and added Cu NP dispersion. The results are summarized in Table 1.

**Table 1:** Samples with various processing steps and the resulting sheet resistance.

Sample #	Description	Sheet resistance [Ω/sq]
1	PEDOT:PSS 1 layer (2000 rpm)	1 M
2	PEDOT:PSS 1 layer (1500 rpm)	230 k
3	PEDOT:PSS 2 layers (1500 + 2000 rpm)	90 k
4	PEDOT:PSS 2 layers (3000 rpm)	85 k
5	PEDOT:PSS 2 layers (1500 rpm)	80 k
6	PEDOT:PSS 3 layers (1500 rpm)	60 k
7	PEDOT:PSS 3 layers (3000 rpm)	28 k
8	PEDOT:PSS 3 layers (1500, 1500, 3000 rpm)	12 k
9	PEDOT:PSS 3 layers (1500, 2000, 3000 rpm)	18 k
10	PEDOT:PSS 3 layers (1500, 3000, 1500 rpm)	21 k
11	PEDOT:PSS 1 layer + Cu(25 nm) 0.2 mg/ml @ 2000 rpm	1.2 k
12	PEDOT:PSS 2 layers + Cu(25 nm) 0.2 mg/ml @ 2000 rpm	467
13	PEDOT:PSS 2 layers + Cu(25 nm) 0.5 mg/ml @ 2000 rpm	459
14	PEDOT:PSS layer + Cu(25 nm) 0.2 mg/ml @ 2000 rpm + PEDOT:PSS layer	1.2 k
15	PEDOT:PSS 2 layers + Cu(60 nm) 0.2 mg/ml @ 2000 rpm	390
16	PEDOT:PSS 3 layers + Cu(60 nm) 0.5 mg/ml @ 1500 rpm	173
17	PEDOT:PSS 3 layers + 3xCu(60 nm) 0.5 mg/ml @ 1500 rpm PEDOT:PSS and Cu @ 1500 rpm	162
18	3x[PEDOT:PSS layer + Cu(60 nm) 0.5 mg/ml @ 1500 rpm]	1 k
19	PEDOT:PSS 3 layers (1500, 1500, 3000 rpm) + 1 × Cu(60 nm) 0.5 mg/ml	200
20	PEDOT:PSS 3 layers (1500, 3000, 1500 rpm) + 1 × Cu(60 nm) 0.5 mg/ml	261
21	PEDOT:PSS 3 layers (1500, 2000, 3000 rpm) + 1×Cu(60 nm) 0.5 mg/ml	256
22	PEDOT:PSS 4 layers (1500, 2000, 3000, 3000 rpm)	4.8K
23	PEDOT:PSS 5 layers (1500, 2000, 3000, 3000, 3000 rpm)	2.3K
24	PEDOT:PSS 6 layers (1500, 2000, 3000, 3000, 3000, 3000 rpm)	972
25	PEDOT:PSS 6 layers (1500, 2000, 3000, 3000, 3000, 3000 rpm) + Cu (60 nm) 0.5 mg/ml	62

The process steps to enhance electrical conductivity are discussed in the text.

**Figure 4:**
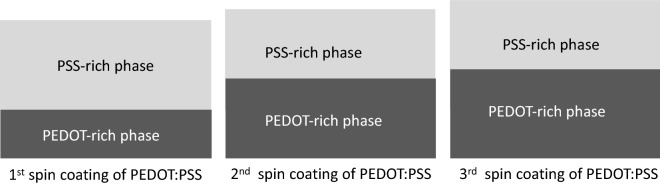
Explanation of how multiple PEDOT:PSS coatings enhance the electrical conductivity of the film. As explained in the text, the thicknesses of a double layer, and a triple layer increase only very slightly over the thickness of a single layer. The multiple coatings help to supplant the PSS top portion of the layer with PEDOT:PSS ribbons.

The doubling of the PEDOT:PSS layer alone decreases the sheet resistance by more than tenfold, from 1MΩ/sq (sample 1) to 90kΩ/sq (sample 3) and 85kΩ/sq (sample 4) depending on the spinning speed. A deposition of a third PEDOT:PSS layer reduced the sheet resistance further to 60kΩ/sq (sample 6) and even to 28kΩ/sq (sample 7) depending on the spinning speed. Even more surprising is the finding that the thickness of the PEDOT:PSS coated twice (twice the entire coating cycle including the annealing step) and thrice does not change the final thickness appreciably. The thickness of a triple deposition at 1500 rpm, 3000 rpm and 1500 rpm gives a final thickness of 85 nm. If the depositions would be strictly additive it would be 65 + 29 + 65 = 159 nm. Of course the spinning speed impacts the final thickness. Thus, for example a triple PEDOT:PSS deposition with consecutively higher speeds: 1500 rpm, 2000 rpm, and 3000 rpm gives a total film thickness of 72 nm, which is only 7 nm thicker than a single layer PEDOT:PSS deposited at 1500 rpm and far less than the additive number of 65 + 56 + 29 = 150 nm. As mentioned in the introduction during the spinning a vertical dephasing of the PEDOT and PSS components takes place. The wet film contains the conductive PEDOT strips at the bottom of the film and a PSS-rich solution at the upper part. During the subsequent soft bake at 120 °C these segregated layers have insufficient time for remixing. The explanation for the phenomenon is given in Fig. 4, where the PSS-rich top layer and the PEDOT-rich bottom layer are shown in a schematically simplified way. There is, of course, no sharp transition between the two phases, but a gradual one, with high concentration of PSS at the top and lowest at the bottom with the reverse behavior of the PEDOT. As pointed out in the introduction, during the spin deposition of PEDOT:PSS the hydrophobic PEDOT-rich phase settles at the bottom of the film and the hydrophilic PSS-rich phase at the top of the PEDOT:PSS film. During the second spin coating the hydrophilic PSS top portion of the film gets exposed to water and is partially supplanted by PEDOT-rich phase.

The same happens during the third spin-coating (samples 6–10) with further lowering of the sheet resistance to 12kΩ/sq (sample 8). As a result, the PEDOT-rich phase grows at the expense of the PSS-rich phase. As the conductive PEDOT-rich phase increases after repeated coating cycles, the electrical conductivity of the PEDOT:PSS film is significantly improved. During the second and third spin coating the wet film helps remove the PSS material and supplant it with conductive PEDOT phase. Consequently, the conductivity increases significantly. The spinning speed has an interesting effect on the conductivity of the PEDOT:PSS films. Sample 8 differs from sample 6 only in the spinning speed of the 3^rd^ PEDOT:PSS layer of 3000 rpm instead of 1500 rpm. The higher speed during the third PEDOT:PSS deposition lowers the sheet resistance by a factor of 5 from 60kΩ/sq to 12kΩ/sq. The explanation for this effect is consistent with explanation given in Fig. 4. The higher speed of the 3rd deposition does not decrease the thickness of the PEDOT:PSS film because the thickness of the overall film is given chiefly by the first coating while the subsequent depositions serve only to densify the PEDOT phase at the bottom portion of the layer forcing this increased connectivity between the PEDOT ribbons.

To verify this assumption, the sheet resistance of a single PEDOT:PSS layer spun at 1500 rpm has been found to be 230kΩ/sq, (sample 2) i.e. 77% lower than the one with PEDOT:PSS spun at 2000 rpm (sample 1). Two layers of PEDOT:PSS both at spun at 3000 rpm (sample 4) resulted in 85kΩ/sq roughly in the same range (80kΩ/sq) as 2 layers spun at 1500 rpm (sample 5). Three layers of PEDOT:PSS (sample 7), with all three coatings spun at 3000 rpm, result in R_sq_ = 28kΩ/sq. This demonstrates a tradeoff between the more denser phase of PEDOT attained at higher speed rates and the thickness of the layer. The denser PEDOT phase decreases R_sq_ while a thinner PEDOT:PSS layer tends to increase R_sq_. Hence, the optimum solution consists in taking advantage of both factors by spinning the first two PEDOT:PSS layers at a lower rate to safeguard a thicker polymer film and add a third layer spun at higher speed to reach higher densification of the PEDOT phase. Samples 8, 9, and 10 show further combinations of the spinning speed combinations. Sample 9 with a sequence of spinning speeds (1500 rpm, 2000 rpm, 3000 rpm) shows a sheet resistance of 18kΩ/sq. Sample 10 with the spinning speed combination (1500 rpm, 3000 rpm, 1500 rpm) yields a sheet resistance of 21kΩ/sq. Sample 8, 9, and 10 show that it is advantageous to perform the first two depositions at low speed to provide a thicker PEDOT:PSS layer and use high speed during the third deposition to attain high PEDOT densification at the bottom of the layer.

To see the impact of multiple coatings, we have increased the number of sequential PEDOT:PSS coatings to six coatings, such that the sample with *n* coatings shares exactly the same (n-1) PEDOT:PSS layers as the sample with (n-1) coatings. In this particular sequence, the 1 × layer PEDOT:PSS sample is sample 2 deposited at 1500 rpm, the second sample is sample 3 with two layers deposited at 1500 rpm and 2000 rpm, respectively. Each additional PEDOT:PSS coating has been deposited at 3000 rpm to maximize the conductivity. The samples with 4 × PEDOT:PSS, 5 × PEDOT:PSS, and 6 × PEDOT:PSS are listed in Table 1 as sample 22, 23, 24, respectively.

**Figure 5:**
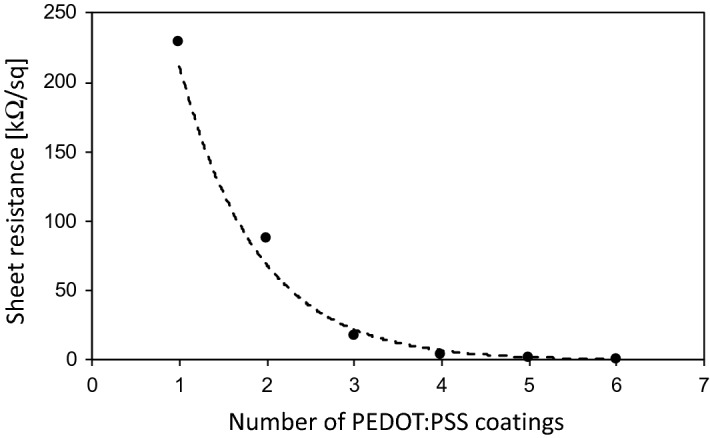
The sheet resistance, R_sq_, of the multiple PEDOT:PSS coatings as a functions of the number of coatings, n. An exponential dependence between R_sq_(n) and n is found and can be expressed as R_sq_(n) = 230 exp[− (n − 1) 1.28] kΩ.

The results of the sheet resistance for the multiple coatings are given in Table 2 and plotted in Fig. 5.

**Table 2:** Sheet resistance R_sq_ versus number of PEDOT:PSS (P/S) coatings.

# P/S layers	1	2	3	4	5	6
R_sq_ in kΩ	230	90	18	4.8	2.3	0.98

From Fig. 5 an exponential dependence of the sheet resistance as function of the number of PEDOT:PSS coatings has been found. The exponential trendline in Fig. 5 can be fitted by the following expression R_sq_(n) = 230 kΩ × exp[− (n − 1) × 1.28]. Thus, it can be seen that further PEDOT:PSS coatings would provide diminishing returns. We will revisit the multiple coatings of PEDOT:PSS in the context of the efficiency of doping by Cu NP of multiple PEDOT:PSS coatings in reducing the overall sheet resistance.

In our approach to add Cu NP to the film we have chosen to add Cu NP at the top after the soft-bake of the spin-coated PEDOT:PSS. In contrast to Ag and Au NPs^30,32^, which can be added to the PEDOT:PSS dispersion and spin coated together with it, the dispersion of Cu NP is difficult and ineffective^46^ because Cu is easily oxidized at ambient temperatures. In fact, our experiments confirmed this assessment. After mixing 0.5 mg/ml Cu(60 nm) NP with PEDOT:PSS in the same aqueous solution to deposit a single PEDOT:PSS layer doped with Cu NP, the sheet resistance of one PEDOT:PSS layer doped in such a way with Cu NP, increased slightly from 1MΩ/sq to 1.2 MΩ/sq. (This sample is not being listed in the Table 1, because such step increases the sheet resistance instead of lowering it). When incorporated Cu NP in an aqueous solution together with PEDOT:PSS, the oxidized Cu NPs cause even more isolation between the PEDOT ribbons. However, when spinning the PEDOT:PSS dispersion with the Cu NPs three times, the sheet resistance of the triple PEDOT:PSS(Cu NP) dropped to 44.4KΩ/sq. (This sample is also not listed in the Table 1.). This result indicates that some of the Cu NP managed to avoid oxidation and enhanced the conductivity by helping bridge electrically the PEDOT ribbons. Of course, such problems would have been avoided, when instead of Cu NP, Au or Ag NPs would have been used. Still, since Cu NPs are more economical and more desirable because of their proven ReRAM switching properties, experiments have been continued with Cu NP being now dispersed in ethylene and deposited on top (to avoid any contact with moisture) of an annealed PEDOT:PSS.

Table 1 shows that the addition of dispersion of Cu NP at the top of the PEDOT:PSS further dramatically increases the conductivity of the film. A single layer of PEDOT:PSS with 0.2 mg/ml Cu(25 nm) (sample11) lowers the sheet resistance from 1MΩ/sq to 1.2kΩ/sq. The combination of 2 layers of PEDOT:PSS with Cu deposited last (sample 12) gives a sheet resistance of 467 and 390 Ω/sq for 25 nm sized and 60 nm-sized Cu NP (sample 15), respectively, indicating that a larger size of Cu NPs is more effective in lowering the sheet resistance. A combination of 3 layers of PEDOT:PSS and with a top layer of Cu NP (60 nm) (sample 16) yields 173 Ω/sq. As can be seen from sample 17, the additional two Cu NP coatings resulted in a marginal improvement from 173 to 162 Ω/sq. Sample 18 is similar to the sample 17, however in sample 17 the Cu dispersion on top of the three layers of PEDOT:PSS has been coated three times with Cu NP. In samples 14 and 18 we investigated the effect of Cu NP coating after deposition of every PEDOT:PSS layer. The resulting sheet resistance is rather high of 1.2kΩ/sq and 1.0kΩ/sq, respectively. The likely reason for the high resistance is that the efficacy of the Cu NP doping due to the Cu NP deposition on the first two PEDOT:PSS layers is degraded by the oxidation when aqueous PEDOT:PSS is being deposited on top of the Cu NP coating. It can, thus, be seen that the effects of multiple spin coatings of PEDOT:PSS and of Cu NP are by no means additive and depend sensitively on the sequence of coatings. In some cases they work synergistically, but in other combinations they can be detrimental to the lowering of sheet resistance.

**Figure 6:**
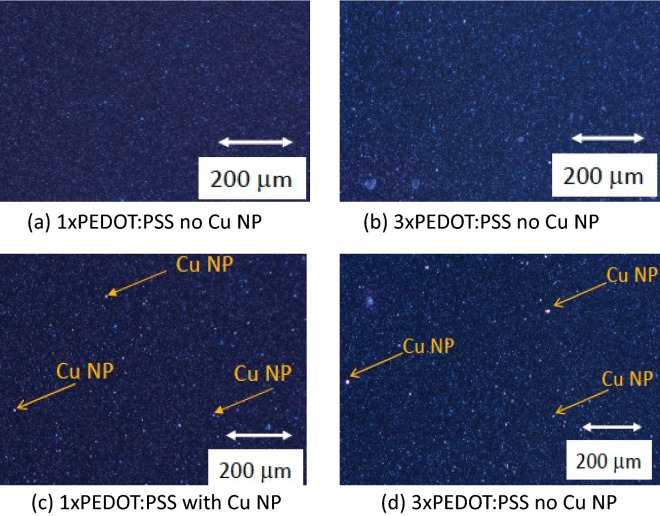
Optical microscope picture of PEDOT:PSS with and without Cu NP. (**a**) single coating of PEDOT:PSS deposited at 1500 rpm with no Cu NP. (**b**) a triple coating of PEDOT:PSS doped with 2 mg/ml of Cu NP. (**c**) a single coating of PEDOT:PSS deposited at 1500 rpm with Cu NP. (**d**) a triple coating of PEDOT:PSS deposited at 1500 rpm with 0.5 mg/ml of Cu NP.

The multiple coatings of PEDOT:PSS, however, appear to be beneficial in all circumstances envisioned here, since they increase the volume of the PEDOT-rich bottom layer and provide a larger potential for conductivity enhancement by metal NP doping, including the Cu NPs. At the same time, due to the nature of particle diffusion, the highest concentration of Cu NPs remains at the top portion of the PEDOT:PSS film that is initially the most resistive part of the film and rendering it significantly more conductive as shown in Fig. 6.

In Table 3 the interaction between the number of PEDOT:PSS coatings and doping with Cu NP and its impact on R_sq_ is elucidated.

**Table 3:** R_sq_ in units of kΩ for multiple coatings with and without Cu NP doping.

# P/S layers	No Cu NP	w/Cu NP	Red. fact
1	230	60	3.8
3	18	0.173	104
6	0.98	0.062	15.8

The reduction factor (Red. Fact.) of R_sq_ by the Cu NP is also indicated.

It can be seen that the relative reduction due to addition of Cu NP is strongest for 3 PEDOT:PSS layer, where the reduction factor is more than two orders of magnitude. In case of 6 PEDOT:PSS layers the reduction factor is only one order of magnitude, whereas the reduction factor for a single PEDOT:PSS layer is less than 4. This particular behavior will become clear when the morphology of the doped and undoped PEDOT:PSS films with multiple coatings will be discussed further below.

The presence of Cu NP at the top of the PEDOT:PSS has the added advantage of making Cu^+^ available for the resistive switching behavior as observed in many ReRAMs including in Cu/TaO_x_/Pt^47^ and Cu/P3HT(GNP)/Au^48^ memory cells. As seen from Table 1 larger size of Cu NP, 60 nm vs 25 nm, helps also to enhance the electric conductivity of the film. Samples 19, 20, and 21 (samples with three PEDOT:PSS layers with Cu (60 nm) NP deposition) and samples 8, 9, and 10, also with three PEDOT:PSS layers with no Cu deposition show similar trends with respect to the spinning speeds of the PEDOT:PSS depositions, confirming that lower spinning speed of the first two depositions followed by a high speed deposition of the third layer yield the lowest sheet resistance.

The synergistic effect of multiple coatings, Cu dispersion and the concomitant soft-bakes can be further optimized to attain even higher conductivities of the film. From Table 1 it can be also seen that the concentration of Cu NP dispersion has a slight enhancement on the enhancement of the conductivity. The comparison of 0.2 mg/ml (sample 12) and 0.5 mg/ml (sample 13) shows that higher concentration of Cu NP in the dispersion leads to a slightly lower sheet resistance.

Since the method of Cu NP doping as a surface layer in conjunction with the multiple PEDOT:PSS coatings proved to be very effective, it was attempted to further optimize the Cu deposition process. In Table 2 we show the results of samples with a triple PEDOT:PSS layer and different Cu NP deposition recipes with subsequent soft bakes at various temperatures are shown. For samples 26–29 the spinning speed of the Cu NP dispersion solution has been lowered from 1500 to 500 rpm in the attempt to increase the thickness of the Cu dispersion coating and thus the Cu concentration on top of the 3^rd^ PEDOT:PSS layer. Then the quadruple layer systems have been annealed between room temperature and 120 °C.

From Table 4 it can be seen that the change of the sheet resistance as function of soft bake at temperatures from 60 °C to 120 °C is insignificant. The soft bake at 60 °C appeared to improve the sheet resistance only slightly, but the subsequent soft bakes at 90 °C and 120 °C degraded the sheet resistance by 6% and 21%, respectively.

**Table 4:** Impact of Cu NP deposition parameters and subsequent soft bake on the sheet resistance of the film.

Sample #	Description	Sheet resistance in Ω/sq
26	PEDOT:PSS 3 layers + Cu(60 nm) 500 rpm; no soft bake	189
27	PEDOT:PSS 3 layers + Cu(60 nm) 500 rpm; soft bake @ 60 °C 10 min	184
28	PEDOT:PSS 3 layers + Cu(60 nm) 500 rpm; soft bake @ 95 °C 10 min	195
29	PEDOT:PSS 3 layers + Cu(60 nm) 500 rpm; soft bake @ 120 °C 10 min	234

Comparing samples 4 and 5 with two PEDOT:PSS layers it can be seen that the maximum spinning speed had a slight effect on the conductivity: the conductivity for the maximum speed of 3000 rpm for both PEDOT layers is 85 kΩ/sq and rotating both layers at 1500 rpm resulted in 80 kΩ/sq, while rotating the first PEDOT:PSS layer at 1500 rpm and the second at 2000 rpm resulted in a sheet resistance of 90 kΩ/sq (sample 3). Two PEDOT:PSS layers at 2000 rpm (not listed in the Table 1) has a sheet resistance of 99.7 kΩ/sq. This result indicates that the sheet resistance increases with the spinning speed caused by the thinning of PEDOT:PSS layer with higher spinning speeds.

The two most promising approaches, i.e. the double PEDOT layer and doping with Cu NP were combined in two separate configurations. In the first configuration, the Cu NP were spun after the deposition of the first PEDOT layer at 2000 rpm. and the second configuration consisted in a double layer PEDOT:PSS deposition, followed by another deposition of the Cu NP. The first configuration yielded a sheet resistance of 1.2KΩ/sq (sample 11) and the second resulted in the very low sheet resistance of only 467 Ω/sq (sample 12).

### Morphology of the PEDOT:PSS films

To characterize the PEDOT:PSS films morphologically optical microscope pictures of the PEDOT:PSS films for multiple coatings have been taken with and without Cu NP as well as Atomic Force Microscopy (AFM) to assess the surface roughness of the films. In Fig. 6 the optical microscope pictures of 1-coating, and 3-coatings of PEDOT:PSS films with and without Cu NP are shown. It can be seen that multiple coatings increase the density of PEDOT:PSS particles seen in the pictures as small white dots. The triple coating of PEDOT:PSS has a more pronounced whitish tint compared with the single PEDOT:PSS coating, indicating a higher density of the PEDOT:PSS composites. The single PEDOT:PSS layer displays wider gaps between the PEDOT:PSS particles rendering the optical image contrast darker. In case of PEDOT:PSS films doped with Cu NP one can distinguish only part of them recognizable by an orange-reddish glow. They can be easily spotted because of the their relatively large size between 60 and 80 nm. If one tilts the sample only so slightly under the microscope, other Cu NP can be seen, when the orientation of their facets cause visible reflection of the light into the camera. The Cu NP appear to settle in places where the density of PEDOT:PSS particles is low. Thus, they tend to fall in the cavities between the PEDOT:PSS particles.

**Figure 7:**
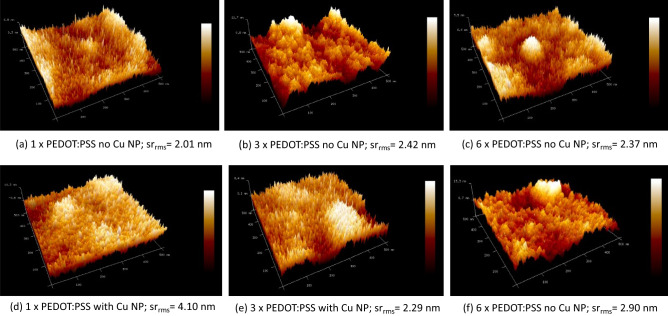
AFM pictures of PEDOT:PSS of a single, triple, and sixfold coatings of PEDOT:PSS with and without Cu NP. The scanned area of all the plots is 500 nm × 500 nm. The root mean square surface roughness, sr_rms_, is also indicated for the respective surface surface. (**a**) a single PEDOT:PSS no Cu NP; sr_rms_ = 2.01 nm, (**b**) a triple PEDOT:PSS no Cu NP; sr_rms_ = 2.42 nm, (**c**) a sixfold PEDOT:PSS no Cu NP; sr_rms_ = 2.37 nm, (**d**) a single PEDOT:PSS with Cu NP; sr_rms_ = 4.10 nm (**e**) a triple PEDOT:PSS with Cu NP; sr_rms_ = 2.29 nm, (**f**) a sixfold PEDOT:PSS with Cu NP; sr_rms_ = 2.90 nm.

Additional information can be gained from AFM pictures of the surfaces of the corresponding PEDOT:PSS films. Of course, the AFM pictures are insensitive to the material differences. In Fig. 7, AFM pictures of selected PEDOT:PSS films are shown. In Table 5, the root mean square (rms) surface roughness of the AFM plots for the respective samples, has been summarized.

**Table 5:** rms surface roughness (SR) in [nm] of PEDOT:PSS films with multiple coatings with and without Cu NP doping.

PEDOT:PSS sample	No Cu NP	w/ Cu NPO
1 × PEDOT:PSS	2.01	4.10
3 × PEDOT:PSS	2.42	2.29
6 × PEDOT:PSS	2.37	2.90

It can be seen that for one coating of PEDOT:PSS the surface roughness with Cu NP is much higher than without Cu NP. This is plausible since the film thickness of 1xPEDOT is ca. 65 nm and the size of Cu NP is between 60 and 80 nm. Thus, inevitably, some Cu NP are bound to stick out, contributing to a higher surface roughness. Nevertheless, the relative low density of PEDOT:PSS particles are able to accommodate the Cu NP is the empty spaces between them as only a small upper part of the Cu NP is sticking out. In case of 3xPEDOT:PSS the film thickness is 85–90 nm and the density of PEDOT:PSS still low enough allowing sufficient free space to accommodate the Cu NP entirely in the film. From Table 5 it can be seen that the surface roughness with Cu NP is now lower than without them (in contrast to the case with one coating and six coatings). The lower surface roughness with Cu NP is likely to be caused by the fact that now the PEDOT:PSS film thickness is larger than the largest Cu NP and the density of PEDOT:PSS composite particles is low enough to accommodate Cu NP entirely within the film. In case of 6xPEDOT, the film thickness is higher than for 3xPEDOT:PSS but the density of PEDOT:PSS is now so high that there are fewer empty spaces to accommodate the Cu NP, thus the surface roughness with Cu NP is somewhat higher than without them (see Table 5). The results in Table 5 correlate perfectly with the findings of the sheet resistance’s dependence on the number of PEDOT:PSS coatings and the reduction factor of the sheet resistance due to the addition of Cu NP, shown in Table 3. The Cu NP have the strongest, relative, impact when the film thickness is equal or larger than the size of Cu NP and the film can offer enough empty space between the PEDOT:PSS particles to accommodate the Cu NP entirely between them.

In addition to the doping techniques demonstrated here, other promising enhancement methods include doping of PEDOT:PSS with Au or Ag NPs or treatment of PEDOT:PSS with acids such as HNO_3_, H_2_SO_4_. The optimum combination will depend on the integration issues of PEDOT:PSS electrodes with possible PEDOT:PSS switching layers into a final organic ReRAM cell. Although the manufacturing of high conductivity of the films is an important objective, the switching performance of a purely organic ReRAM memory cell is even more important, rendering the process integration and optimum combination of enhancement techniques, a non-trivial task.

## Summary

We have presented new methods of enhancing the electrical conductivity of PEDOT:PSS layers. It was shown that multiple spin coatings of PEDOT:PSS by itself can lower the sheet resistance by more than two orders of magnitude, while not appreciably increasing the total thickness of the layer. It was found that the spinning speed of every coating plays a sensitive role, where the first coating should be deposited at a lower speed to establish the thickness of the final PEDOT:PSS layer. The subsequent coatings should be done at higher speeds which, apparently, lead to the growth and densification of the PEDOT ribbons at the bottom of the layer. An exponential dependence between the sheet resistance and the number of PEDOT:PSS coatings has been demonstrated. Despite negative findings on the use of doping by Cu NP^46^, Cu NP doping proved a viable conductivity enhancement technique, provided that it is deposited on an already baked PEDOT:PSS film, rather than pre-mixing the Cu NP with the aqueous dispersion of PEDOT:PSS. Coating the surface of PEDOT:PSS with Cu NP can result in reduction in the sheet resistance by four orders of magnitude. Higher concentrations and larger sizes of Cu NP help further decrease the sheet resistance, albeit not substantially. It was also demonstrated that these high conductivity PEDOT:PSS films can be patterned both on oxidized Si wafers and flexible Mylar substrate using sacrificial metal layer to protect PEDOT:PSS from the damage caused by the photolithography process.

Present results may be combined with already known techniques to further increase the conductivity of PEDOT:PSS such as acid treatment (H_2_SO_4_, H_3_PO_4_, or H_3_BrO_3_) and the doping of PEDOT:PSS with Au and Ag NPs. These options will be reported in a future work.

## Data Availability

All data generated or analyzed during this study are included in this published article.
